# A dataset of genetic diversity studies in the China Seas

**DOI:** 10.1038/s41597-024-03082-w

**Published:** 2024-02-23

**Authors:** Yaqian Ming, Gang Ni

**Affiliations:** Ministry of Education Key Laboratory of Mariculture, Ocean University of China, Qingdao, 266003 China

**Keywords:** Population genetics, Biodiversity

## Abstract

Genetic diversity, a fundamental aspect of biodiversity, greatly influences the ecological and evolutionary characteristics of populations and species. Compiling genetic data is crucial as the initial step in comprehending and applying genetic resources; however, regional collating work is still insufficient, especially in marine ecosystems. Here, by conducting a thorough literature search and quality-control procedures, we provide a dataset of genetic diversity studies on marine species in the China Seas. The final dataset comprised a total of 746 studies (encompassing 840 data sets and 3658 populations) across 343 species from 1998 to 2022. For each data set, information including publication year, publication language, studied species, belonged taxonomic group, applied molecular markers, and sampling strategies (number of populations, total number of individuals, etc.) was collated to analyse the scope, strengths, and omissions of these works. This dataset offers a comprehensive overview of genetic diversity studies in the China Seas, which may help to adjust future research focuses, promote conservation and macrogenetics studies in this region, and also facilitate regional cooperation.

## Background & Summary

Genetic diversity, defined as the variety of alleles and genotypes within a population^1^, represents the most fundamental level of biodiversity. Genetic diversity provides the raw materials for species’ evolution and adaptation, closely related to ecosystem processes and human well-being^2–5^. A dataset of genetic diversity data is the initial step to achieve the purposes above. Our knowledge regarding comprehensive genetic diversity studies remains insufficient. To date, most performed studies focused on terrestrial species^6–10^, with only several performed in the marine realm^11–13^.

The China Seas, with their diverse climate zones, ecosystems, and coastal currents, boast one of the world’s richest marine biodiversity^14^. While comprehensive collating work at the species level had been conducted in the China Seas since the early 1950s^15^, our understanding of the scope and focus of genetic diversity studies is still limited.

In the past two decades, the genetic diversity of marine species in the China Seas has been extensively investigated using a range of molecular markers, thanks to advancements in population genetics and phylogeography. The majority of these studies primarily focused on small-scale geographical populations of one or a few species, and solely evaluated their genetic diversity, thus lacking a comprehensive summary^16^. The integration of these empirical studies, considering the taxonomic molecular markers and geographic coverage, helps to inform the scope, strengths, and omissions of these works, which is crucial to adjust future goals and directions in time.

Here we provide a comprehensive compilation of studies published on the population genetics of species in the China Seas. After conducting a literature search and quality-control procedures, the dataset consisted of 746 published studies and 840 data sets, including 3658 populations across 343 species. We found that (1) Publications per year: The number of publications per year increased steadily from 1998 until 2012 and reached a plateau recently. (2) Taxonomic groups studied: Fish, mollusk, and crustacean groups were the most representative taxonomic groups, accounting for 46.43%, 23.45%, and 14.76%, respectively. (3) Molecular markers applied: According to the six classes of molecular markers we defined, Class I (mtDNA) was predominantly applied in all studies (47.14%) and also in most taxonomic groups, while the highly praised Class IV (SNP) accounted for a small proportion (4.05%). (4) Sampling effort: Unbalanced geographic sampling was evident at both provincial and prefecture-city levels, and an insufficient number of collected populations was also suggested. The dataset provides a basis for future synthetic work in the China Seas, which may help to promote studies on conservation genetics and macrogenetics in this region.

## Methods

### Data collection

**Fig. 1:**
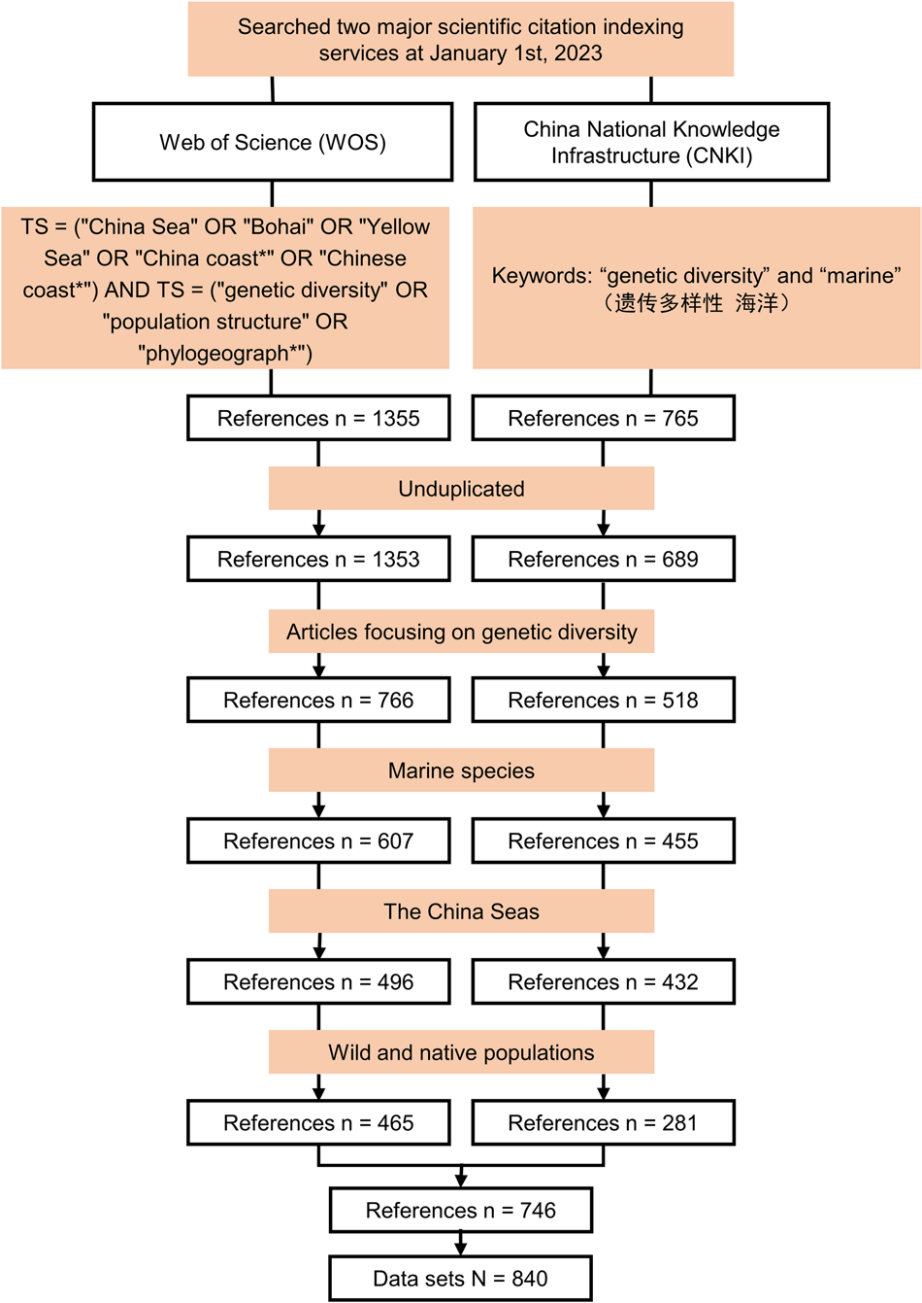
An overview of the procedures and results of literature search and filtering.

The procedure and results were shown in Fig. 1. A literature search was conducted using two major scientific citation indexing services, the Web of Science (WOS) (http://apps.webofknowledge.com/) and the China National Knowledge Infrastructure (CNKI) (http://www.cnki.net/), which can cover both Chinese and English peer-reviewed literature^17^. We searched the WOS database using the search rule TS = (“China Sea” OR “Bohai” OR “Yellow Sea” OR “China coast*“ OR “Chinese coast*“) AND TS = (“genetic diversity” OR “population structure” OR “phylogeograph*“) on 1st January 2023, and searched the CNKI database using the two keywords “genetic diversity” and “marine” (in Chinese).

A study that meets the following four criteria was retained: (1) articles focusing on genetic diversity; (2) marine species; (3) the China Seas; and (4) wild and native populations. Specifically, literature on pure phylogeny, taxonomy, different life history stages of the same species, and single population for validation of primers or markers suitability were discarded; cultured populations, invasive species, parasites, and pathogens were not taken into account in the dataset. To avoid duplicative data sets, references using raw data from other studies were not considered. Once a study was selected, we extracted: the year of publication, scientific nomenclature of studied species, taxonomic groups (i.e. fish, mollusk, crustacean, plant, and other), applied molecular markers and their belonged classes, sampling effort (including the total number of populations, the total number of samples for all populations, the minimum and maximum number of samples among populations, and each sampled location at both provinces and prefectural-level cities).

All of the used molecular markers were divided into the following six classes: Class I, mitochondrial DNA (mtDNA); Class II, nuclear DNA sequences (nrDNA), amplified fragment length polymorphism (AFLP), random amplified polymorphic DNA (RAPD), inter-simple sequence repeat (ISSR), restriction fragment length polymorphism (RFLP), sequence-related amplified polymorphism (SRAP), and restriction site amplified polymorphism (RSAP); Class III, microsatellite (SSR); Class IV, single nucleotide polymorphism (SNP); Class V, isozymes; Class VI, combination (using more than one type of molecular marker).

### Data visualization

To analyse the usage of different marker classes over time, we employed linear regression to model the percentage of data sets in which each marker class was used as a function of the publication year. Linear regression was also used to gain insights into the status of the sampling effort by modelling the total number of samples as a function of the total number of geographic populations based on different taxonomic groups and marker classes.

The sampling sites displayed in the article were directly extracted from provincial and prefectural administrative areas. Counties, towns, or villages mentioned in the articles were identified as prefecture-level cities through the Ministry of Civil Affairs’ national administrative division information query platform (in the year 2023, http://xzqh.mca.gov.cn/map). To avoid inaccuracies, some non-coastal cities were assigned to the provinces instead of the prefectural-level cities.

## Data Records

Data from the dataset can be downloaded as one XLSX file on Figshare^18^. It contains 840 rows and each of the rows represents a data set. The columns contained are as follows:

1. RefID: identifying the references.
2. Year: publish year.
3. Groups: taxonomic groups.
4. Species: identifying the species.
5. Pop_n: the total number of populations.
6. Total_n: the total number of samples for all populations.
7. Min_n: the minimum number of samples among populations.
8. Max_n: the maximum number of samples among populations.
9. Markers: molecular markers used in populations.
10. Classes: classification of molecular markers used in populations.

An additional XLSX file containing not only the 746 corresponding references for each reference ID, but also the 1374 discarded references with reasons from the initial dataset, is also available from Figshare^18^.

## Technical Validation

### Data validity

**Fig. 2:**
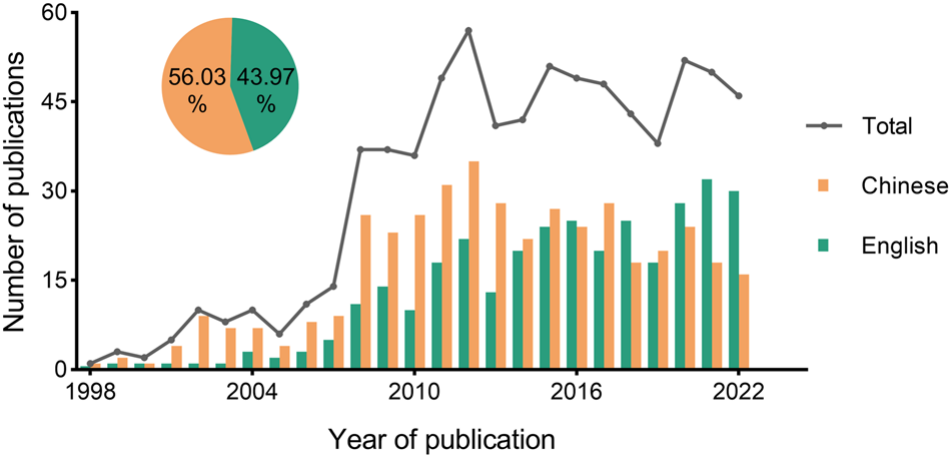
Interannual variation and percentage composition of 746 genetic diversity studies in different languages from 1998 to 2022.

From 1998 to 2022, a total of 746 studies on the genetic diversity of marine species in the China Seas were published (Fig. 2). Of these, more studies were published in Chinese, accounting for 56.03% (418 of 746), while studies published in English accounted for 43.97% (328 of 746). After a period of rapid growth from 1998 until 2012, the number of Chinese, English, and total publications recently reached a plateau. In addition, the relationship between species and studies is many-to-many, resulting in a total of 840 data sets across 343 species. To adhere to the filter criteria and ensure the accuracy of the information, data on taxonomic groups, molecular markers, and geographic information were initially extracted and subsequently subjected to a thorough double-checking process. We standardized the scientific nomenclature for synonyms. For studies in which partial data were cited, we examined the cited studies to ensure the accuracy of the data. Meanwhile, a logic test was conducted to verify the accuracy of the sampled data: Min_n * Pop_n ≤ Total_n ≤ Max_n * Pop_n.

### Taxonomic and molecular markers’ bias

**Fig. 3:**
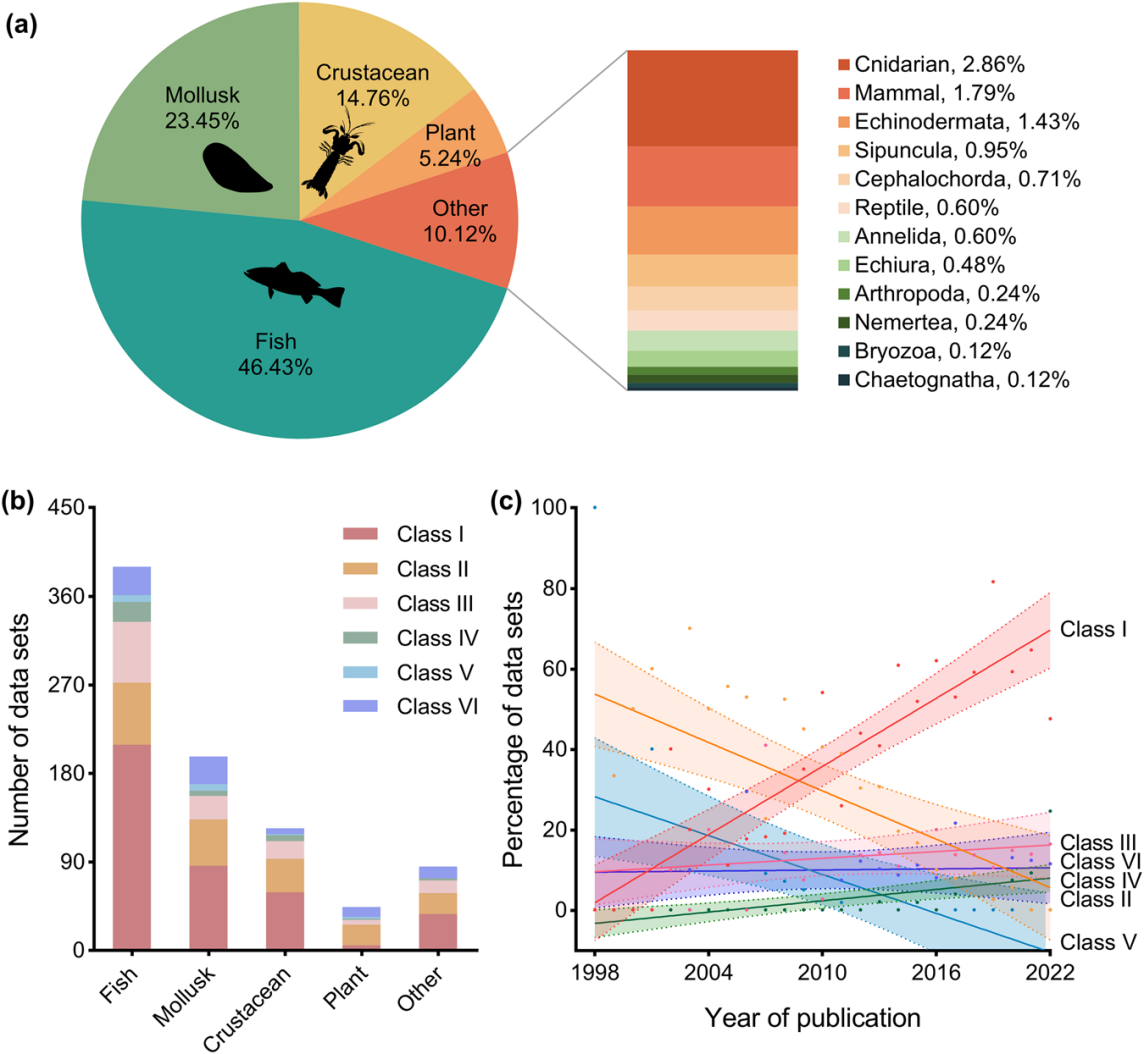
Composition of taxonomic groups and markers classes across 840 data sets from 1998 to 2022. (**a**) The proportion of taxonomic groups. (**b**) The number of data sets of the six marker classes by taxonomic groups. (**c**) Interannual variation on marker classes use. Using linear regression, we modeled the percentage of data sets using each class of markers over the publication years. There was a significant increase in the percentage of data sets that used Class I (*p* < 0.0001) and Class IV (*p* = 0.0006). The percentage of Class II showed a significant decrease over time (*p* = 0.0002), as did the percentage of Class V (*p* = 0.0043). There was no statistically significant difference observed in the percentage of data sets that used Class III (*p* = 0.3396) or Class VI (*p* = 0.8848). Silhouettes were from PhyloPic (http://phylopic.org).

The proportion of data sets showed a tendency of concentration among taxonomic groups (Fig. 3a). From 1998 to 2022, fish was the most representative taxonomic group, accounting for 46.43% (390 of 840) of the data sets, followed by mollusk (23.45% [197 of 840]) and crustacean groups (14.76% [124 of 840]). The proportion of each other taxonomic group (e.g. plant, cnidarian, and mammal) was relatively small (no more than 5.24%). In the fish group, the most recorded species were *Larimichthys polyactis*, *Larimichthys crocea* and *Pampus argenteus*, accounting for 4.87% (19 of 390), 3.33% (13 of 390) and 3.33% (13 of 390), respectively. *Mytilus Coruscus* (5.58% [11 of 197]) and *Ruditapes philippinarum* (5.08% [10 of 124]) had the highest number of data sets in the mollusk group, while *Portunus trituberculatus* (18.55% [23 of 124]) and *Oratosquilla oratoria* (14.52% [18 of 124]) ranked first and second in the crustacean group, respectively. All the species mentioned above are traditionally important economic fishery species in the China Seas.

The composition of molecular markers used by different groups showed a preference for Class I (Fig. 3b). In fish, mollusk, crustacean, and other groups, Class I was the most commonly used molecular marker class. Their usage frequency accounted for 53.59% (209 of 390), 43.65% (86 of 197), 47.58% (59 of 124), and 43.53% (37 of 85) of the data sets for each group, respectively. The observed and expected (based on an average proportion of 1/6) counts of Class I in each group differed significantly (Chi-squared test, *p* < 0.0001 for all the groups above). The popularity of applying mtDNA in population genetics studies can be attributed to its well-known properties, including maternal inheritance, small size, and high mutation rates^19^.

The use of different marker classes changed over the years with three trends (Fig. 3c). There was a significant increase in the percentage of data sets that used Class I (*p* < 0.0001) and Class IV (*p* = 0.0006). The percentage of Class II showed a significant decrease over time (*p* = 0.0002), as did the percentage of Class V (*p* = 0.0043). There was no statistically significant difference observed in the percentage of data sets that used Class III (*p* = 0.3396) or Class VI (*p* = 0.8848). Class I was the most popular and widely used molecular marker type, comprising almost half (47.14% [396 of 840]) of all classes. Although there was an increasing trend, high-resolution Class IV based on Next Generation Sequencing (NGS) accounted for only 4.05% (34 of 840). NGS approaches are well-known for their high resolution and efficiency^20–23^, and we fully expect to see a rise in their application, particularly in the marine realm.

### Unbalanced and insufficient sampling

**Fig. 4:**
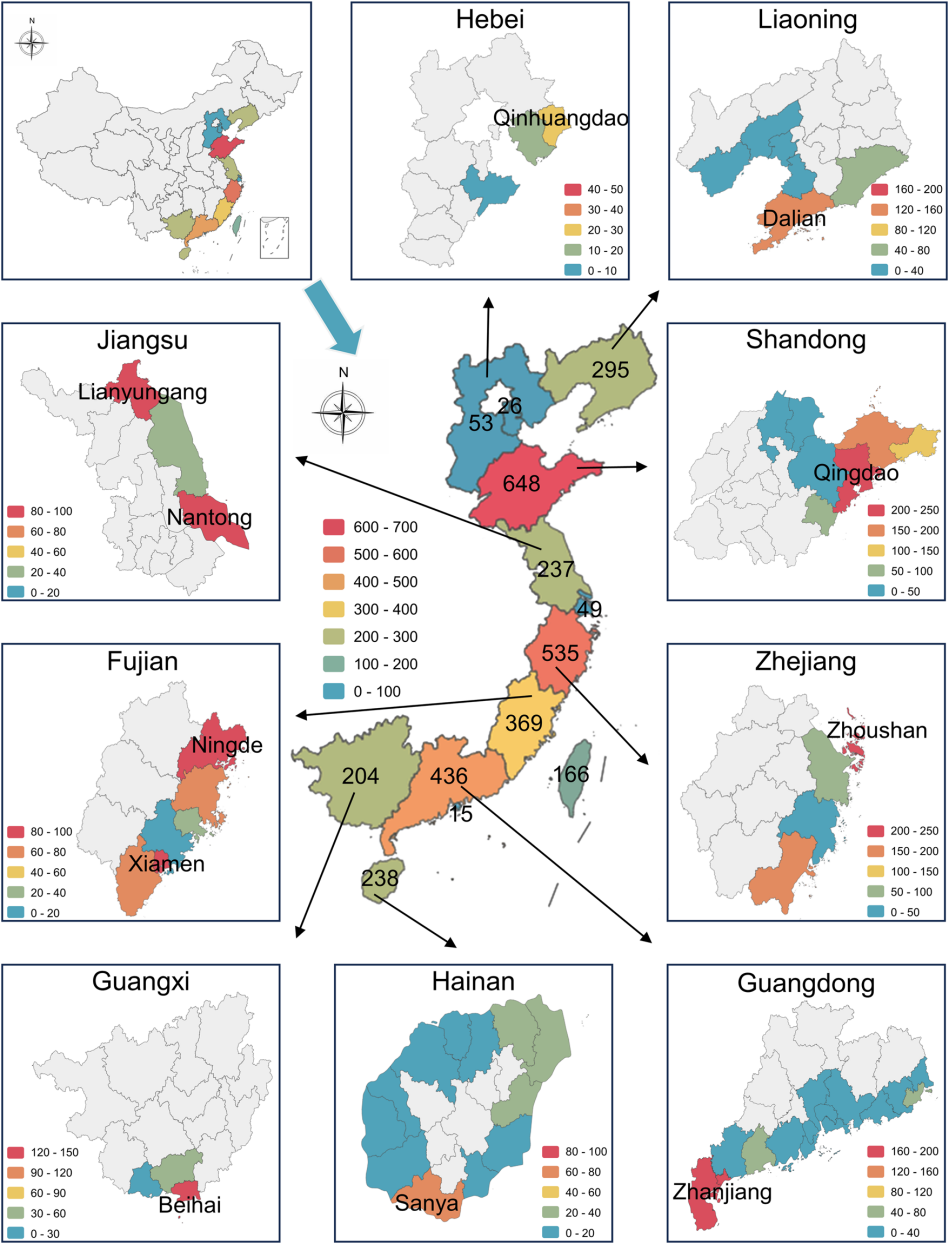
Sampling distribution map of different administrative areas. Nine mainland provinces had prefecture-level city sampling maps. The numbers on the provinces in the middle of the picture represent the number of samples from different regions. Tianjin (26), Shanghai (49), Taiwan (166) and Hong Kong (15) were not shown their specific names.

In the dataset, we identified a total of 3658 sampling sites and pinned the map (Fig. 4). Sampling in both provinces and prefecture-level cities showed an unbalanced trend. The sampling was concentrated in several provinces, the top three sampled provinces were Shandong (17.71% [648 of 3658]), Zhejiang (14.63% [535 of 3658]), and Guangdong (11.92% [436 of 3658]) provinces. Within each province, some cities were found with extremely high sampled populations, such as Beihai (64.22% [131 of 204]) in Guangxi, Qinhuangdao (56.60% [30 of 53]) in Hebei, and Dalian (54.24% [160 of 295]) in Liaoning. This unbalanced sampling pattern is possibly related to the decline of fishery resources and the increase in costs. To save costs, researchers tend to prefer pioneer sites with high sampling frequency, which can serve as anchor sites for taxa and spatio-temporal comparisons^11^.

**Fig. 5:**
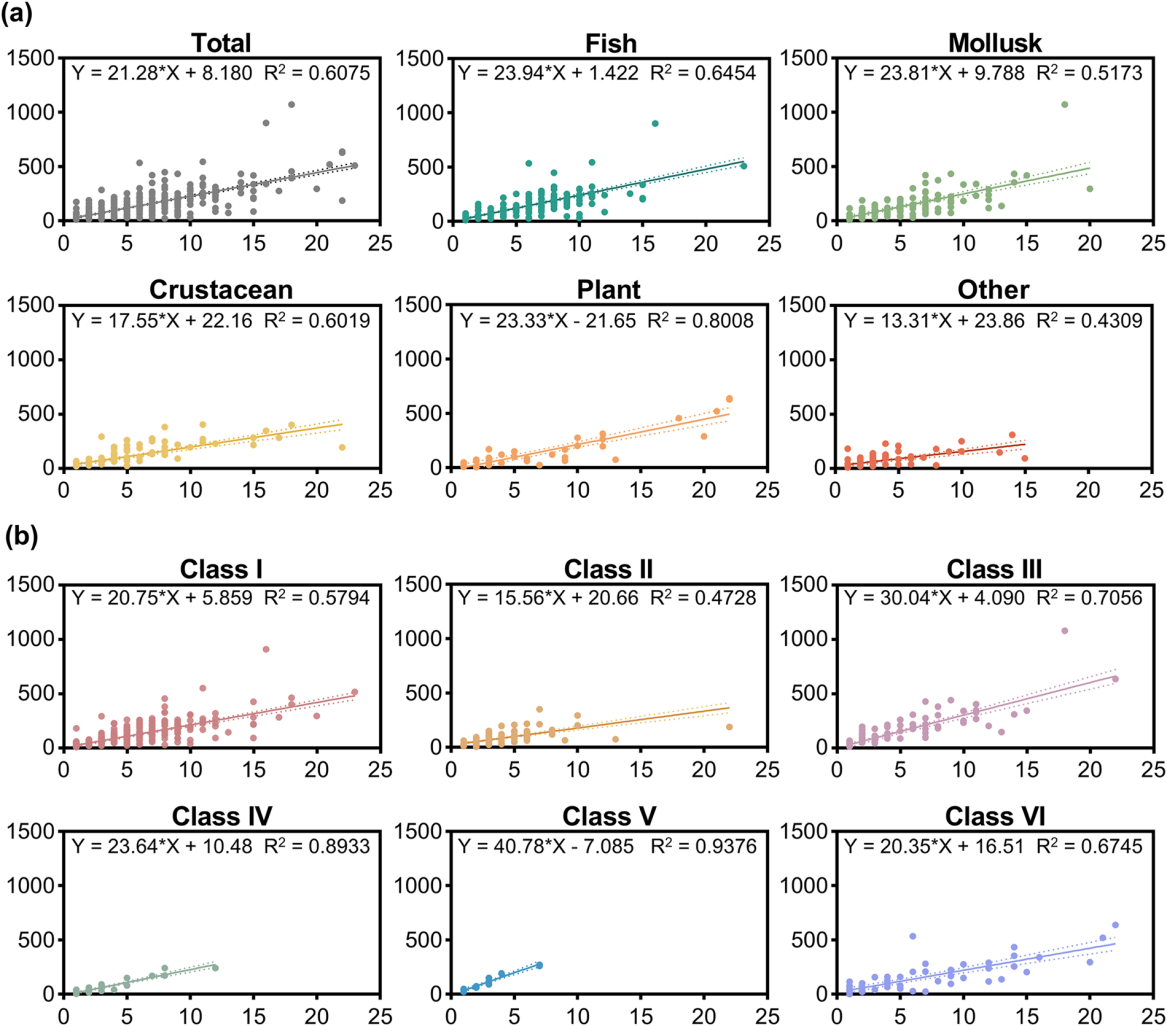
The variation of the total number of samples (Total_n) of different (**a**) taxonomic groups and (**b**) marker classes with the number of geographical populations (Pop_n). Using linear regression, we modeled the total number of samples for different geographic population numbers. The formulas were at the top of the graph.

The sample size is a crucial factor in research design as it relates to research cost and the robustness of conclusions^24^. Given the distinct characteristics of the collected samples, it is crucial to elucidate the current status of sampling. Linear regressions showed the relationship between the number of geographic populations and the total number of samples by taxonomic groups (Fig. 5a) and marker classes (Fig. 5b). However, data sets with only one geographic population comprised 27.73% (233 of 840) of the overall data sets of various species. The high concentration of sampling within three populations (51.55% [433 of 840]) indicated that the wildlife sampling conducted in the China Seas was insufficient. It is conceivable that small or imbalanced sample sizes may obscure temporal trends or spatial genetic variance^25^. The need for strengthening the sampling effort of wild populations in the China Seas persists.

## Usage Notes

There was an overlap between the journals included in Web of Science (WOS) and China National Knowledge Infrastructure (CNKI). Data sets in the WOS database were preserved if they were simultaneously recorded in two databases. This was done because these data sets contain titles, abstracts, and other information presented in English, which enhances their visibility and accessibility.

Criteria (2) and (4) were not combined into the criterion of “wild and native population of marine species”, because each study had its specific filtering criteria. This feature allows users to choose studies according to their specific requirements and preferences.

The dataset was not filtered for the number of geographic populations or the number of samples per geographic population. Meta-analysis studies may apply different filtering criteria according to their research purposes. For instance, it was necessary to have a minimum of three distinct geographic populations and a minimum of five individuals sampled from each population simultaneously^11^. Alternatively, a minimum of three distinct geographic populations and a total of 30 sampled individuals were required^16^. It is possible to further explore genetic patterns after applying stricter filtering criteria.

## Data Availability

There is no custom code produced during the collection and validation of this dataset.
